# Retrograde signaling from functionally heterogeneous plastids

**DOI:** 10.3389/fpls.2012.00286

**Published:** 2012-12-19

**Authors:** Anna Lepistö, Jouni Toivola, Lauri Nikkanen, Eevi Rintamäki

**Affiliations:** Molecular Plant Biology, Department of Biochemistry and Food Chemistry, University of TurkuTurku, Finland

**Keywords:** light signaling, redox signals, nuclear gene expression, stress, differentiation, NTRC

## Abstract

Structural and functional components of chloroplast are encoded by genes localized both to nuclear and plastid genomes of plant cell. Development from etioplasts to chloroplasts is triggered by light receptors that activate the expression of photosynthesis-associated nuclear genes (PhaNGs). In addition to photoreceptor-mediated pathways, retrograde signals from the chloroplast to the nucleus activate or repress the expression of nuclear genes involved in acclimatory or stress responses in plant leaves. A plant mesophyll cell contains up to 100 chloroplasts that function autonomously, raising intriguing questions about homogeneity and coordination of retrograde signals transmitted from chloroplast to nucleus. We have previously demonstrated that the knockout of the chloroplast regulatory protein, chloroplast NADPH-dependent thioredoxin reductase (NTRC) leads to a heterogeneous population of chloroplasts with a range of different functional states. The heterogeneous chloroplast population activates both redox-dependent and undifferentiated plastid-generated retrograde signaling pathways in the mutant leaves. Transcriptome data from the *ntrc* knockout lines suggest that the induction of the redox-dependent signaling pathway depends on light conditions and leads to activation of stress-responsive gene expression. Analysis of mutants in different developmental stages allows to dissect signals from normal and anomalous chloroplasts. Thus, the signals derived from anomalous chloroplasts repress expression of PhaNGs as well as genes associated with light receptor signaling and differentiation of stomata, implying interaction between retrograde pathways and plant development. Analysis of the nuclear gene expression in mutants of retrograde signaling pathways in *ntrc* background would reveal the components that mediate signals generated from heterogeneous plastids to nucleus.

## INTRODUCTION

Light is the primary environmental factor controlling plant development and acclimation processes, regulating the entire life cycle of plants from seed germination to seed production (Sullivan and Deng, 2003). Light is perceived directly by blue (cryptochromes CRY, phototropins, and zeitlupe ZTL) and red light (phytochromes PHY) photoreceptors, which then activate signaling networks to initiate an array of light response processes such as photomorphogenesis, photoperiodic development, as well as acclimatory and protective modifications of plants. Light signals are also mediated by chloroplasts to control chloroplast biogenesis and acclimation to changes in light quality, quantity, and day length. Transcriptomics studies have demonstrated that between 5 and 25% of Arabidopsis (*Arabidopsis thaliana*) genes are light-regulated, depending on gene content in microarrays and experimental conditions (Jiao et al., 2007; Sharrock, 2008; Li et al., 2012). Recently, light receptor-dependent signaling pathways have been suggested to interact with chloroplast retrograde signaling pathways (Ruckle and Larkin, 2009). The mechanisms by which photoreceptor-dependent signals and chloroplast signals interact are not well understood. Here we review recent findings from the study of the light and retrograde signaling pathways and discuss evidence showing interaction of these signaling pathways. We also present a hypothesis proposing that a heterogeneous plastid population leads to formation of distinct retrograde signals from chloroplast to nucleus. The hypothesis is based on our analysis of nuclear gene expression in an Arabidopsis mutant containing both photosynthetically active chloroplasts and non-photosynthetic plastids in a single mesophyll cell.

## LIGHT SIGNALING PATHWAYS IN THE CONTROL OF PHOTOSYNTHETIC DEVELOPMENT OF LEAF

Light receptors control leaf development in angiosperm species by regulating chloroplast biogenesis. Development of chloroplasts from etioplasts is triggered by light by two primary mechanisms. In the absence of light, nuclear repressor molecules such as constitutive photomorphogenic 1 (COP1) and phytochrome-interacting factors (PIFs) cause degradation of positive light regulators that would activate the expression of light-responsive genes, thereby suppressing light-induced processes and maintaining etiolation-specific processes (see the reviews by Bae and Choi, 2008; Bu et al., 2011; Li et al., 2012). Upon illumination, light-activated phytochromes and cryptochromes move from cytoplasm to the nucleus and drive photomorphogenetic development of seedlings by removing repressors from the nucleus and by enhancing the expression of the positive light regulators like HY5 (long hypocotyl 5), and Golden 2-likes (GLKs) proteins (Nagy and Schäfer, 2002; Bae and Choi, 2008; Waters et al., 2009; Bu et al., 2011). The removal of COP1 from the nucleus also stabilizes the positive regulators (Bae and Choi, 2008) which, in turn, activate the transcription of genes involved in chloroplast development, cell division, and plant growth. Expression of light-induced genes was recently found also to be regulated by epigenetic factors (Li et al., 2012). In angiosperms, chlorophyll is synthesized exclusively in light because the reduction of protochlorophyllide to chlorophyllide is energized by photons absorbed by protochlorophyllide bound to the protochlorophyllide oxidoreductase (POR) enzyme (Reinbothe et al., 1996).

Besides light receptor-driven signaling networks, retrograde signals from chloroplast and mitochondria to the nucleus impact seedling development and plant acclimation to environmental cues (Larkin and Ruckle, 2008; Pogson et al., 2008; Woodson and Chory, 2008; Kleine et al., 2009; Inaba, 2010; Jung and Chory, 2010; Barajas-López et al., 2012; Leister, 2012). Retrograde signals can activate or repress nuclear gene expression, depending on the genes and processes dissected. Several sources of retrograde signals in chloroplast have been identified during last decades, including altered production of tetrapyrrole biosynthesis intermediates, defective expression of plastid genes, production of reactive oxygen species (ROS) in plastids, and the redox state of thylakoid electron transfer components (PET; Pfannschmidt et al., 1999; Sullivan and Gray, 1999; Pursiheimo et al., 2001; Strand et al., 2003; Piippo et al., 2006; Pesaresi et al., 2007; Kim et al., 2008; Muhlenbock et al., 2008; Foyer and Noctor, 2009; Lepistö and Rintamäki, 2012). Redox components at the acceptor side of photosystem I (PSI) also initiate retrograde signals that modify nuclear gene expression (Pursiheimo et al., 2001; Piippo et al., 2006).

The routes of retrograde signal transmission within the chloroplast, through the cytoplasm and eventually to the nucleus are still fairly unknown, although some components of the signaling pathway have been identified. A genetic screen for potential signaling molecules identified a number of *gun* (*genomes uncoupled*) mutants in which the nuclear gene expression was unresponsive to plastid signals (Mochizuki et al., 2001). This approach identified the *GUN1* gene encoding a chloroplast pentatricopeptide repeat-containing protein (Koussevitzky et al., 2007). GUN1 has been described as a “switchboard” inside a chloroplast that can receive signals from tetrapyrrole intermediates, chloroplast translation machinery (Koussevitzky et al., 2007; Woodson and Chory, 2008; Cottage et al., 2010), and from the redox state of PET (Inaba, 2010; Sun et al., 2011). Chloroplast proteins EXECUTER 1 and 2 (EX1, EX2) are components of a ^1^O_2_-dependent retrograde signaling route that controls cell death in plants (Wagner et al., 2004; Kim et al., 2008). Recently, highly promising candidates mediating the signal from chloroplast to nucleus has been identified. Phosphoadenosine phosphate (PAP) has been suggested to carry information from chloroplast to nucleus (Estavillo et al., 2011). PAP accumulates in chloroplast in response to drought and high light and moves to nucleus, in which it activates the expression of stress-related genes (Estavillo et al., 2011). Sun et al. (2011) also identified a promising candidate for a mediator of retrograde signal from chloroplast envelope to nucleus. The homeodomain transcription factor PTM is attached to the chloroplast envelope. Following a signal from the chloroplast, a peptide is cleaved from the N-terminus of PTM and the peptide translocates to the nucleus where it activates expression of *ABI4*, a nuclear AP2-type transcription factor. ABI4 was previously shown to act downstream of GUN1 in the plastid-derived signaling pathway and to repress the expression of photosynthetic genes by binding to CCAC motif upstream of light-responsive genes (Koussevitzky et al., 2007). Another nuclear transcription factor, GLK2, has been proposed to act downstream from chloroplast retrograde signaling. GLK1 and GLK2 control chloroplast biogenesis and acclimation of a plant to light intensity by preferentially activating the expression of genes in chlorophyll biosynthesis and light-harvesting complexes (Waters et al., 2009). The expression of both *GLK*s genes is regulated by phytochromes (Tepperman et al., 2006), while the expression of GLK2 also responds to plastid-derived signals (Waters et al., 2009).

## ACCLIMATION OF THE PHOTOSYNTHETIC STRUCTURES TO LIGHT INTENSITY AND TO THE LENGTH OF DIURNAL PHOTOPERIOD

Plants adjust leaf cell morphology and chloroplast ultrastructure according to incident light conditions in order to coordinate absorption of solar energy with the capacity for carbon assimilation. This light acclimation involves adjustments to the photosynthetic apparatus, such as changes in photosystem stoichiometry and the size of light-harvesting antennae, as well as modulation of stromal enzyme activities and antioxidant production (Walters and Horton, 1995; Vanderauwera et al., 2005; Bartoli et al., 2006; Li et al., 2009). Several reports suggest that the light signal triggering the modification of photosynthetic traits is perceived in chloroplast rather than mediated by cytoplasmic light receptors (Pfannschmidt et al., 1999; Pursiheimo et al., 2001; Piippo et al., 2006; Muhlenbock et al., 2008; Bräutigam et al., 2009; Foyer and Noctor, 2009).

In addition to light intensity, the length of the diurnal photoperiod influences on the development of leaf structure and composition of chloroplasts. We have shown that Arabidopsis plants grown under identical light intensities in either short or long photoperiods show both structural and photosynthetic characteristics typical of shade or sun plants, respectively (Lepistö et al., 2009; Lepistö and Rintamäki, 2012). The characteristics modified by the length of the photoperiod include the density of stomata in leaf epidermis, respiration and CO_2_ assimilation capacity, the ultrastructure of chloroplast, and the chlorophyll a/b ratio in thylakoid membranes (Lepistö et al., 2009; Lepistö and Rintamäki, 2012). Thus, the modifications of photosynthetic traits induced by photoperiod length resemble light intensity acclimation strategies. Acclimation of chloroplast ultrastructure to light intensity is largely controlled by chloroplast signals, whereas light receptor signaling associated with the circadian clock regulates the photoperiodic development in plants. The signaling cascade controlling photoperiodic development consists of complex network of multiple, functionally-redundant regulators within a circadian clock (for recent reviews, see Turck et al., 2008; Harmer, 2009; Imaizumi, 2010; Song et al., 2010). The circadian clock is entrained to a 24-h cycle by photoperiodic signals transmitted from photoreceptors, and while the light-regulated mechanisms of resetting the clock are still not clear, expression of components of transcriptional feedback loops within the circadian clock is known to be regulated by light (Imaizumi, 2010; Song et al., 2010). Importantly, interaction between the circadian clock and light receptors is complex, since the circadian clock also controls the adaptation of light signaling pathways to the light/dark cycles (Li et al., 2012). Whether signals generated in chloroplasts also regulate the photoperiodic development of photosynthetic structures in leaves, and whether these signaling pathways are independent or interconnected with guiding leaf differentiation under various light regimes, are interesting questions that remain to be answered.

## MUTATION IN CHLOROPLAST COMPONENTS AS A TOOL TO DISSECT CHLOROPLAST-TO-NUCLEUS RETROGRADE SIGNALING

Chloroplast retrograde signaling pathways have largely been investigated by dissecting nuclear gene expression in the *gun* mutants (Mochizuki et al., 2001). In these studies, norflurazon (NF) and lincomycin treatments that induce bleaching of seedlings have been used to generate signals from non-functional plastids (Mochizuki et al., 2001, 2008; Strand et al., 2003; Moulin et al., 2008; Cottage et al., 2010). It is likely, however, that these harsh treatments induce secondary modifications in nuclear gene expression that confound interpretation of the experimental data. On the other hand, mutating chloroplast proteins to impair chloroplast function without inducing plastid bleaching is also an approach to investigate chloroplast retrograde signaling pathways. Some chloroplast mutants exhibiting conditional phenotype that appear only under specific circumstances (Yu et al., 2007; Kim et al., 2008; Sirpiö et al., 2008; Lepistö et al., 2009; Rosso et al., 2009; Tikkanen et al., 2010) can also be used to dissect signaling pathways.

**FIGURE 1:**
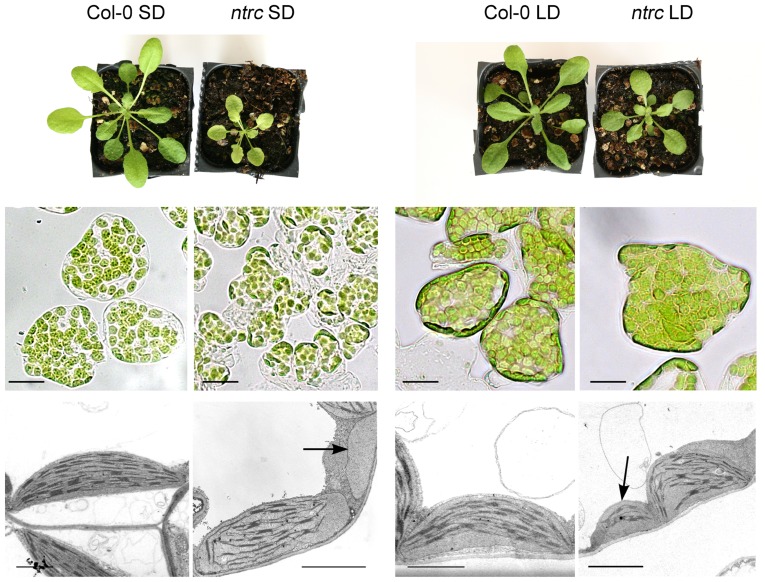
**Rosette phenotypes, bright field images, and electron micrographs of the mesophyll cells in wild-type and *ntrc *line.** The plants were grown under short (SD) and long (LD) photoperiod of 8 h and 16 h light, respectively. Plastid-like organelles with poorly developed thylakoid membranes are indicated by arrows. Scale bars: 20 μm in light microscope images and 2 μm in electron micrographs, respectively.

We have employed an Arabidopsis mutant lacking the nuclear-encoded chloroplast regulatory protein, chloroplast NADPH-dependent thioredoxin reductase (NTRC) to dissect chloroplast retrograde signaling pathway. NTRC is a member of chloroplast thioredoxin family (Serrato et al., 2004). Redox-active cysteines in thioredoxins are used to reduce disulfide bridges in target proteins. NTRC knockout mutants (*ntrc*) have reduced growth and decreased chlorophyll content (Perez-Ruiz et al., 2006; Lepistö et al., 2009), indicating that it is an important component of the chloroplast redox network. NTRC has been shown to regulate the activities of chloroplast proteins involved in ROS scavenging, and in the synthesis of chlorophyll, starch, and aromatic amino acids (Perez-Ruiz et al., 2006; Stenbaek et al., 2008; Kirchsteiger et al., 2009; Lepistö et al., 2009; Michalska et al., 2009; Pulido et al., 2010). Intriguingly, *ntrc* mutants possess both normal chloroplasts and irregularly differentiated plastids in a single mesophyll cell (**Figure 1**; Lepistö and Rintamäki, 2012). Some of the chloroplasts in *ntrc* are elongated and possess anomalous terminal appendages (Lepistö, 2011). The mesophyll cells of *ntrc* lines also contain small plastid-like organelles with poorly developed thylakoid membranes (**Figure 1**; Lepistö and Rintamäki, 2012), suggesting that NTRC controls early steps of chloroplast differentiation.

The phenotype of the *ntrc* mutant depends on light conditions (Perez-Ruiz et al., 2006; Lepistö et al., 2009), and is most pronounced when plants are grown under short photoperiods (**Figure 1**), especially under high light. On the other hand, low light and long photoperiods reduce growth defects in *ntrc* lines. In comparison to wild-type, 60 and 90% retardation of the *ntrc* biomass was recorded under long and short photoperiod, respectively (Lepistö et al., 2009). The anomalous *ntrc* chloroplasts were present in seedlings as well as in young developing and mature leaves grown under all light conditions studied (**Figure 1**; Lepistö, 2011), suggesting that generally slow growth of *ntrc* plants is primarily due to the defects in chloroplast differentiation in the absence of NTRC. It is likely, however, that the further reduced growth rate under short photoperiods is caused by imbalance in starch metabolism that is more severe in *ntrc* mutants grown under a shorter photoperiod (Lepistö, 2011). Defective starch metabolism (Kirchsteiger et al., 2009; Lepistö, 2011) impaired the utilization of light energy for carbon fixation in *ntrc* lines acclimated to short photoperiod, thereby increasing the reduced state of the components in PET. Accordingly, *ntrc* leaf grown under short photoperiod suffered from chronic photoinhibition of PSII in growth light (Lepistö et al., 2009).

## TWO MODELS FOR RETROGRADE SIGNALING PATHWAYS IN *ntrc* KNOCKOUT LINES

**FIGURE 2:**
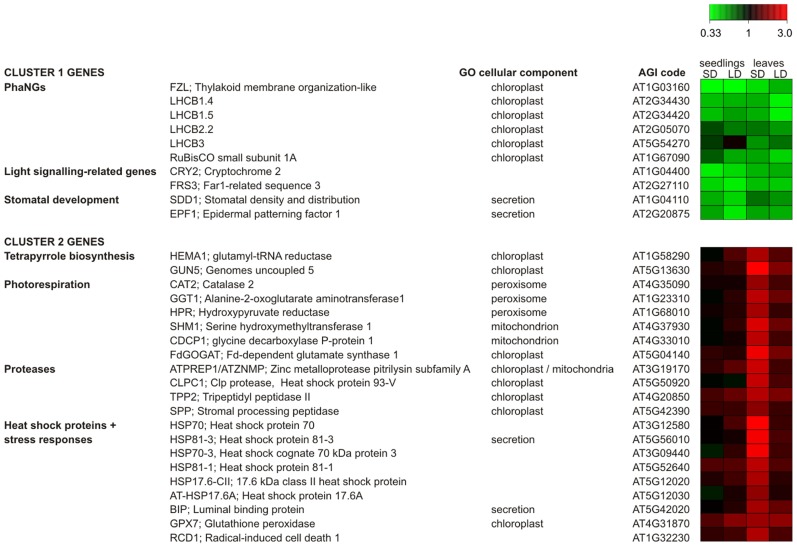
**Differentially expressed cluster 1 and cluster 2 genes in *ntrc* relative to wild-type *Arabidopsis* in 10-d-old seedlings and rosette leaves.** The plants were grown under short (SD) and long (LD) photoperiod of 8 and 16 h light, respectively. Values are the means ± SEM of three independent biological replicates. For standard errors, *p*-values and for a complete list of differentially expressed genes (see Lepistö et al., 2009, Supplemental Table S2 online; www.plantphysiol.org). Copyright American Society of Plant Biologists.

The *ntrc* lines are valuable in dissecting different aspects of chloroplast-to-nucleus retrograde signaling pathways by (i) showing how heterogeneous population of plastids in a single cell influences the quantity, quality, and complexity of chloroplast signals and (ii) facilitating the study of conditionally induced retrograde signals in chloroplast. Genome-wide transcript profiling of *ntrc* lines revealed two gene expression clusters in mutant plants (**Figure 2**; Lepistö et al., 2009). The first cluster contained genes that were repressed in *ntrc *independently of photoperiod length and leaf age, including photosynthetic genes, light signaling genes, and the genes regulating the stomatal density in leaf epidermis (cluster 1 in **Figure 2**). The hypocotyl of *ntrc* lines has a weakened response to far-red and low fluence-rate blue light (Lepistö et al., 2009) that is coincident with the repression of the CRY2 gene and a component of the far-red light signaling pathway, respectively (**Figure 2**). Furthermore, the *ntrc* lines also have reduced ability to control the stomatal density under light conditions in which the differentiation of epidermal cells to guard cells is reduced in wild-type leaves (Lepistö et al., 2009). Accordingly, the expression of the genes encoding the repressors of the development of stomatal guard cells (SDD1 and EPF1) is significantly reduced in *ntrc* lines (**Figure 2**). Another 60 genes were also repressed in *ntrc* lines independently of the age or growth light conditions (Lepistö et al., 2009). Half of these repressed genes encode unknown proteins or proteins with putative domains, while the rest of the repressed genes cannot be categorized to any specific functional groups or linked to visible *ntrc* phenotype.

Because NTRC is a chloroplast-localized protein, the down-regulation of cluster 1 genes is likely due to a signal from *ntrc *chloroplast to nucleus. These results show that this repressive chloroplast signal not only down-regulates photosynthetic genes, but also controls processes linked to photosynthetic function such as stomatal differentiation. Furthermore, down-regulation of genes responsive to far-red light and low fluence-rate blue light, along with the long hypocotyl phenotype in the mutant, indicate that the chloroplast signal in *ntrc* interacts with signaling pathways controlled by light receptors.

The second cluster contained genes that were conditionally up-regulated in mature leaves of *ntrc *plants (cluster 2 in **Figure 2**), with stronger expression levels coinciding with a stronger *ntrc *mutant phenotype. The cluster 2 includes genes of chlorophyll synthesis that are strongly light-regulated (Matsumoto et al., 2004). In addition, cluster 2 genes encode enzymes in the photorespiration pathway, as well as chloroplast proteases and several heat shock proteins that are involved in stress responses (**Figure 2**). Another 30 genes (Lepistö et al., 2009) show expression profile similar to cluster 2 genes in **Figure 2**. Interestingly, cluster 2 genes were not up-regulated in young *ntrc* seedlings indicating that the regulatory signal generated from the chloroplast may arise from long-term modifications of chloroplast metabolism.

**FIGURE 3:**
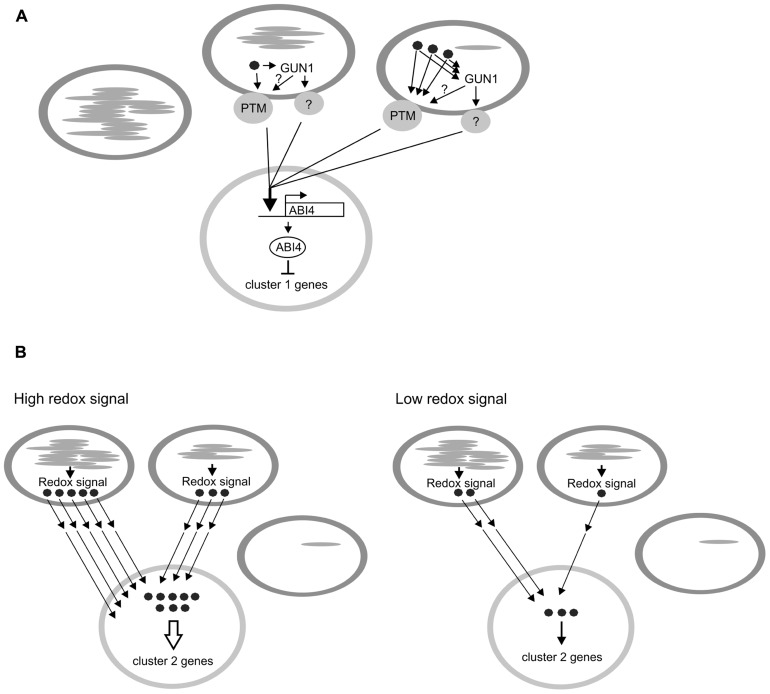
**Models for the plastid-to-nucleus retrograde signaling pathway initiated from plastids in *ntrc* mesophyll cell.** **(A)** Signal (•) derived from anomalous plastids in *ntrc* leaves. This signal is mediated by GUN1 and/or PTM to nucleus, where the N-terminal fragment of PTM induces the *ABI4* expression. ABI4, in turn, represses the expression of cluster 1 genes (**Figure 2**). The expression level of cluster 1 genes does not correlate with the abundance of the signal originally generated in the plastids. **(B)** Redox-dependent retrograde signaling pathway in *ntrc* mesophyll cell. Redox signal (•) is conditionally generated in *ntrc* chloroplast containing functional thylakoids. The abundance of the signal is high in chloroplasts with low capacity to utilize absorbed light energy in carbon fixation. The signal exits from chloroplast and interacts with the downstream component(s) in cytosol or in nucleus, where the expression of cluster 2 genes is activated. For details, see the text.

Light conditions have a different effect on the expression of the clusters 1 and 2 genes in *ntrc *lines, suggesting that retrograde signals initiate at different sources. Can these signals be identified and how are they transduced from chloroplasts to the nucleus? Repression of cluster 1 gene expression resembles the expression pattern of genes in treatments abolishing plastid function or plastid gene expression (Sullivan and Gray, 1999; Strand et al., 2003; Koussevitzky et al., 2007; Ruckle et al., 2007; Mochizuki et al., 2008). This retrograde signal is therefore likely to be a result of poorly differentiated anomalous chloroplasts in *ntrc *mesophyll cells (**Figure 1**). We hypothesize that the poorly differentiated small plastids arise from asymmetric division of a chloroplast in an expanding *ntrc* leaf (Lepistö, 2011). The irregular division may result in unequal distribution of resources between daughter plastids that impairs the development of the smaller plastid. Anomalous chloroplasts are present in *ntrc* cotyledons and leaves grown under various light conditions and the abundance even rises in expanded leaves (**Table 1**). However, cluster 1 genes were equally down-regulated in seedlings and mature leaves of *ntrc*, and their repressed expression was unrelated to the severity of the mutant phenotype, indicating that the regulation of cluster 1 genes does not depend on the abundance of anomalous chloroplasts. This suggests that the regulatory effect is independent of the strength of retrograde signals that are emitted from these plastids. The plastid signal is probably detected by a downstream signaling component inside the chloroplast or in the envelope, which relays the information through the cytoplasm to the nucleus (see the scenario in Figures 1C and 2 in Leister, 2012), where a nuclear component of the signaling cascade activates expression of the repressor, which in turn controls the expression of target genes (**Figure 3A**). The chloroplast retrograde signaling pathway recruiting GUN1 and/or PTM fulfills the criteria for retrograde signaling pathway repressing the cluster 1 genes in *ntrc* (**Figure 3A**). Both signaling components have shown to act downstream to chloroplast signal and up-stream to ABI4, a repressor of light-induced genes. The knockout lines of *gun1* and *ptm* under standard growth conditions are indistinguishable from wild-type (Mochizuki et al., 2001; Sun et al., 2011). Testing the nuclear gene expression in *ntrc* mutants in *gun *and *ptm* backgrounds under various light conditions would reveal whether GUN1 and/or PTM mediates a signal generated from an anomalous *ntrc *plastid to nucleus.

**Table 1:** The leaf width, the area of palisade mesophyll cells, and the number of chloroplasts in Col-0 and *ntrc* grown under short day (SD) and long day (LD) condition.

**Growth conditions and age**		**Leaf number**	**Leaf width (mm)**	**Palisade mesophyll cell area (μm^2^)**	**Chloroplasts per palisade mesophyll cell transection**	**Chloroplasts per 100 μm^2^ of palisade cell area**	**Chloroplasts per 100 μm^2^ of palisade cell area (% of Col-0)**
SD,	Col-0	1	2.9 ± 0.2	358 ± 12	7.5 ± 0.2	2.09	
10 days	*ntrc*	1	1.7 ± 0.3	199 ± 8	4.4 ± 0.2	2.21	105%
SD,	Col-0	7	9.4 ± 1.3	1008 ± 40	9.9 ± 0.2	0.98	
4 weeks	*ntrc*	7	4.7 ± 0.3	684 ± 29	4.9 ± 0.2	0.72	73%
SD,	Col-0	12	11.2 ± 0.9	771 ± 32	9.7 ± 0.3	1.28	
6 weeks	*ntrc*	12	7.2 ± 1.2	857 ± 33	6.8 ± 0.2	0.79	61%
LD,	Col-0	6	9.4 ± 1.9	1564 ± 60	10.3 ± 0.3	0.66	
3 weeks	*ntrc*	6	7.2 ± 0.6	1788 ± 72	10.1 ± 0.3	0.56	84%

*Data are determined from light microscope cross-sections of leaf (see Lepistö and Rintamäki, 2012). The parameters measured for SD plants with different age indicate that in comparison to wild-type the relative proportion of chloroplasts with differentiated thylakoids decreases as the *ntrc* palisade cell and leaf expands. The decrease likely depends on the accumulation of small plastids with poorly developed thylakoids in *ntrc* cells, which are not visible in light microscope cross-sections of leaf. Data are presented as means ± SEM of 30 cells in four independent experiments (leaf width, palisade mesophyll cell area, and chloroplast number per mesophyll cell).*

We propose that the expression of the cluster 2 genes in *ntrc* is regulated by a different signaling pathway than the one described for cluster 1 genes. The transcript levels of the up-regulated cluster 2 genes in *ntrc *lines were positively correlated with the severity of the mutant phenotype. The short photoperiod that induced the strongest mutant phenotype in *ntrc* also significantly enhanced photoinhibition of PSII in mutant *ntrc* leaves (Lepistö et al., 2009). The short photoperiod also caused a severe imbalance in starch metabolism (Lepistö, 2011) that decreases the utilization of light energy and consequentially increases the redox status of chloroplasts (Lepistö et al., 2009). Thus, the signal activating the expression of cluster 2 genes in mature *ntrc* chloroplast may arise from reduced components of the electron transfer chain, likely from the plastoquinone pool or from the acceptor side of PSI (Pfannschmidt et al., 1999; Pursiheimo et al., 2001; Piippo et al., 2006; Pesaresi et al., 2007; Bräutigam et al., 2009; Barajas-López et al., 2012). This redox signal activates expression of genes involved in stress responses, such as heat shock proteins and chloroplast proteases. Photorespiratory genes also respond to this redox signal, likely because photorespiration has been proposed to protect chloroplasts against over-reduction by dissipating excess light energy that cannot be utilized in photosynthetic carbon metabolism (Kozaki and Takeba, 1996). Cluster 2 genes are only slightly up-regulated in *ntrc* plants acclimated to a long photoperiod because of fewer redox signals are produced in the photosynthetically active chloroplasts with less attenuated starch metabolism (**Figure 3B**; Lepistö, 2011).

Expression of *HEMA1 *and *GUN5*, members of the most important light-regulated gene cluster in tetrapyrrole synthesis (Matsumoto et al., 2004), was also conditionally up-regulated in *ntrc* leaves (**Figure 2**). Heme and intermediates of chlorophyll biosynthesis are thought to act as signaling molecules in the chloroplast-derived signaling pathway (Strand et al., 2003; Woodson et al., 2011). In comparison to wild-type, *ntrc* lines accumulated higher amount of the chlorophyll biosynthesis intermediate magnesium protoporphyrin IX (Mg-Proto; Stenbaek et al., 2008). Therefore, tetrapyrrole biosynthesis intermediates may mediate and/or strengthen the redox signal generated by light reactions in *ntrc* lines. Tetrapyrrole intermediates are reported to generate signals repressing photosynthesis-associated nuclear genes (PhaNG) expression (Woodson and Chory, 2008; Inaba, 2010), but this has been subsequently challenged (Mochizuki et al., 2008; Moulin et al., 2008). On the other hand, Mg-Proto and heme have been shown to stimulate *HSP70* and *HEMA *gene expression in *Chlamydomonas *(Vasileuskaya et al., 2004; von Gromoff et al., 2006, 2008), which resembles the response observed in *ntrc *leaves. The heme- and Mg-Proto-dependent signaling cascade in *Chlamydomonas* differs significantly from the GUN1-mediated pathway (von Gromoff et al., 2008), suggesting that this signaling route is GUN1-independent, although nuclear factor(s) involved in heme- or Mg-Proto-dependent signaling are not known (von Gromoff et al., 2008). With respect to the signal characteristic, conditionally induced retrograde signal in *ntrc* leaves (**Figure 3B**) resembles the passive diffusion transport mechanism described by Leister (2012) in Figure 1C. In this scenario, the chloroplast signal migrates from the chloroplast to the cytoplasm and/or to the nucleus, in which the expression level of cluster 2 genes depends on the concentration of signaling molecule (**Figure 3B**). To find components of this signaling pathway, *ntrc* lines can be transformed with a reporter gene fused to the promoter of cluster 2 genes and subsequently mutagenizing these transgenic lines by ethyl methane sulfonate (EMS). Mutants that no longer respond to the conditional chloroplast signal would potentially contain mutations in signaling components of this pathway.

## Conflict of Interest Statement

The authors declare that the research was conducted in the absence of any commercial or financial relationships that could be construed as a potential conflict of interest.
